# Progress on the UNTANGLE Challenge for revealing cooperative motions

**DOI:** 10.1063/4.0001085

**Published:** 2025-10-27

**Authors:** James M Holton

**Affiliations:** UCSF, San Francisco, CA, USA

## Abstract

Since announcing the UNTANGLE Challenge last year several ground-breaking new algorithms have emerged to meet it. I will review these new tools and discuss their efficacy, both with the challenge data and how they can make real-world structures better. The Challenge itself will also be evaluated and updated, based on experience and feedback from the community. The Challenge posits that cooperative motions of functional dynamics can be extracted from ordinary, average electron density data using nothing more than established geometry restraint information. It consists of a simple 2-state system with 11 levels of increasing difficulty. The model with the best score reflects the true, underlying cooperative motion of the system, and the more untangled the model gets it both becomes closer it is to this true ensemble and it also becomes easier to detect the tangles. A full solution has yet to be found, but great strides have been made. In the near future, untangling multi-conformer models will lead to better geometry scores and lower R factors, and also reveal more detail in electron density maps as well as more biologically relevant cooperative motions.

